# Stent-assisted coil embolization of ruptured vertebral artery dissected aneurysm with severe stenosis of bilateral vertebral artery V4 segment by the transmountain technique: a case report and review of the literatures

**DOI:** 10.3389/fsurg.2025.1442122

**Published:** 2025-02-17

**Authors:** Guangzhi Hao, Zijun Zhang, Yuwei Han, Yu Huan, Yushu Dong, Haiyang Zhao, Guobiao Liang

**Affiliations:** ^1^ Department of Neurosurgery, General Hospital of Northern Theater Command, Shenyang, China; ^2^ Department of Neurosurgery, Lingyuan Central Hospital, Lingyuan, Liaoning, China

**Keywords:** vertebral artery dissecting aneurysm, subarachnoid hemorrhage, stent-assisted coil embolization, intravascular reconstruction, stent assist technology

## Abstract

A 46-year-old woman presented with acute head and neck pain for 10 h. Head CT showed subarachnoid hemorrhage (SAH) and digital subtraction angiography (DSA) identified a ruptured dissected aneurysm of the right vertebral artery with severe artery stenosis. Moreover, an unruptured dissecting aneurysm and severe vascular stenosis were also found in the left vertebral artery. How to deal with ruptured bleeding aneurysm and prophylactically deal with contralateral unruptured dissecting aneurysm and the stenosis of the vertebral artery has become a thorny problem. By adopting the Transmountain technique, we used a single Enterprise-2 stent to cover the neck of the right ruptured vertebral artery dissection aneurysm and the severe stenosis of the distal vessel, while bypassing the vertebrobasilar artery junction to continue covering the severe stenosis and the unruptured dilated dissection of the contralateral vertebral artery. This new stent-assisted approach may provide a reference for clinicians in the treatment of complex dissection aneurysms.

## 1 Introduction

Vertebral artery dissecting aneurysm (VADA) are becoming the more common cause of spontaneous subarachnoid hemorrhage (SAH) in young and middle-aged people (1). The rebleeding rate of ruptured intracranial VADA was high and the prognosis was very poor (2). Previous studies have shown that the rate of rebleeding in VADA was as high as 70%, the rate of rebleeding within 24 h was more than 50%, and the fatality rate was as high as 46% (3, 4). Intracranial vertebral artery had thin outer vascular membrane, few elastic fibers in the media, and lack of surrounding soft tissue support, which was more prone to bleeding (5, 6). Most of the bleeding was located in the posterior cranial fossa and the fourth ventricle, which could compress the brain stem, and was often accompanied by respiratory dysfunction and life-threatening (7). Clinically, once the diagnosis of vertebral artery dissection aneurysm was clear, effective and active treatment should be taken as soon as possible to reduce the risk of aneurysm re-rupture, so as to reduce the mortality or disability rate.

Here, we reported a novel treatment procedure for a particular complex case of ruptured vertebral artery dissecting aneurysm. By using a single Enterprise-2 stent and a small number of coils, we not only completely embolized the ruptured aneurysm and improved the ipsilateral severe stenosis, but also prophylactically alleviated the contralateral vertebral artery dissection and stenosis.

## 2 Material and method

### 2.1 Eligibility criteria and search strategy

The screening process of the literature review was shown in Supplement Figure S1, and finally we identified five related articles describing VADA that are similar to ours, as shown in Table 1 (8–12). Inclusion criteria were as follows: (1) Vertebrobasilar artery aneurysms were clearly identified after cerebral vascular digital subtraction angiography. (2) The patient has detailed disease data and follow-up information. (3) The age of the patient is over 10 years old. The exclusion criteria were as follows: (1) Cases in which no treatment was given included surgery and medication. (2) Cases undergoing surgery cases in which follow-up information or surgical information is missing.

**Table 1:** Summary of cases of vertebral artery dissected aneurysm reported in the literature.

Author, year	Age, sex	Symptom	Maximum size (mm)	Neck diameter (mm)	Location	Treatment	Complications	Results	Follow-up (month)	Clinical outcome
Takahiro Yokoyama, 2024	40, male	Mild headache	Not described	Not described	Left VA	Proximal clipping	Right vertebral artery expansion	CO	6	Improved
L. Liu, 2010	30, male	Headache and dizziness	9.7	5.3	Basilar Trunk	SAT by Leo stent	None	CO	6	Improved
Yukihiko Nakamura, 2023	67, male	Impaired consciousness	12.6	6	left VA	Coil embolization	Cerebral vasospasm	CO	2	Gradually recovered
MohammedA. M., 2023	12, male	Diplopia, vomiting, ataxia, and severe headache	Not described	Not described	Left VA at the C3 and C4 levels	Oral aspirin	None	Periodic review	2	Improved
S. Angiafico, 2003	57, female	Acute excruciating headache	Not described	Not described	V4 segment of the left VA	Coil embolization	Ischemic stroke	CO	6	Gradually recovered

CO, complete occlusion; SAT, stent-assisted technique; VA, vertebral artery.

### 2.2 Selection process and data collection process

Our team has three independent personnel to conduct literature search on pubmed, web of science, sciencedirect and other websites. After being reviewed by additional staff, the appropriate data is finally integrated.

### 2.3 Study risk of bias assessment and reporting bias assessment

We used the RoB 2.0 tool to assess the risk of bias in the included studies. Minimize the risk of sample selection bias and measurement bias. Independent staff rechecked the publication status of all included studies and confirmed that no relevant published studies had been missed. At the same time, the findings were fully reported to avoid the possibility of reporting bias.

## 3 Case report

This study was approved by the Ethics Committee of Lingyuan Central Hospital. The patient and family members signed a written informed consent to the surgery and the publication of the case report, and all the imaging information is anonymized.

**Figure 1:**
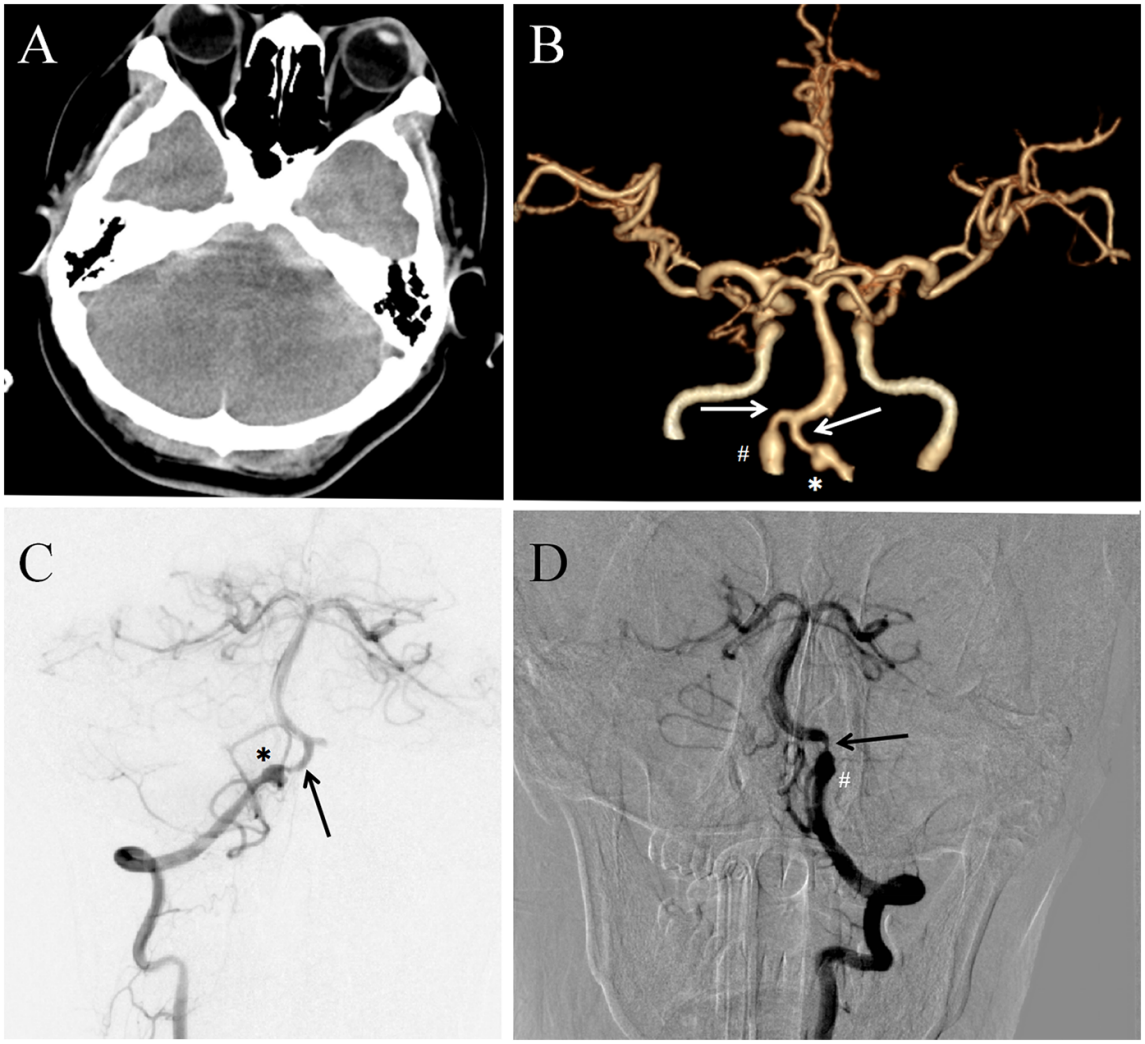
**(A)** Preoperative computed tomography of the head showed subarachnoid hemorrhage. **(B)** CT angiography (CTA) suggested a right ruptured vertebral artery dissection aneurysm marked by “*”. “#” indicated an unruptured dilated dissection of the left vertebral artery. Two arrows indicated severe stenosis at the ends of both vertebral arteries. **(C)** Digital subtraction angiography (DSA) identified a dissected aneurysm of the right vertebral artery with severe stenosis of the distal vessel. **(D)** DSA also showed an unruptured dissecting aneurysm and severe vascular stenosis in the left vertebral artery.

A 46-year-old woman was admitted to the local hospital on May 14, 2024 with sudden severe headache and neck pain. The patient had a history of hypertension for 4 years and was treated with telmisartan orally. The patient denied the history of diabetes, heart disease, hepatitis, tuberculosis, mental illness, trauma and blood transfusion. Physical examination revealed lethargy, stiff neck, unresponsiveness and no paralysis. Preoperative head CT showed subarachnoid hemorrhage (Figure 1A), which was mainly concentrated in the pontine cistern and the cistern of pontocerebellar angle. Preoperative emergency CTA revealed an irregular-shaped aneurysm of the right vertebral artery with a severe stenosis (Figure 1B). After admission, the patient underwent further digital subtraction angiography (DSA) examination. Right vertebral arteriography revealed an irregular ruptured aneurysm in the V4 segment of the right vertebral artery with a size of about 5.5 mm × 4.0 mm and a neck of about 3.0 mm (Figure 1C). In addition, it was found that the right vertebral artery terminal was severely narrowed, with a stenosis degree of 80% (Figure 1C). The stenosis of the left vertebral artery terminal segment could reach 90%, and the local dilation of the left vertebral artery V4 segment indicated the presence of an unruptured dissecting aneurysm (Figure 1D).

**Figure 2:**
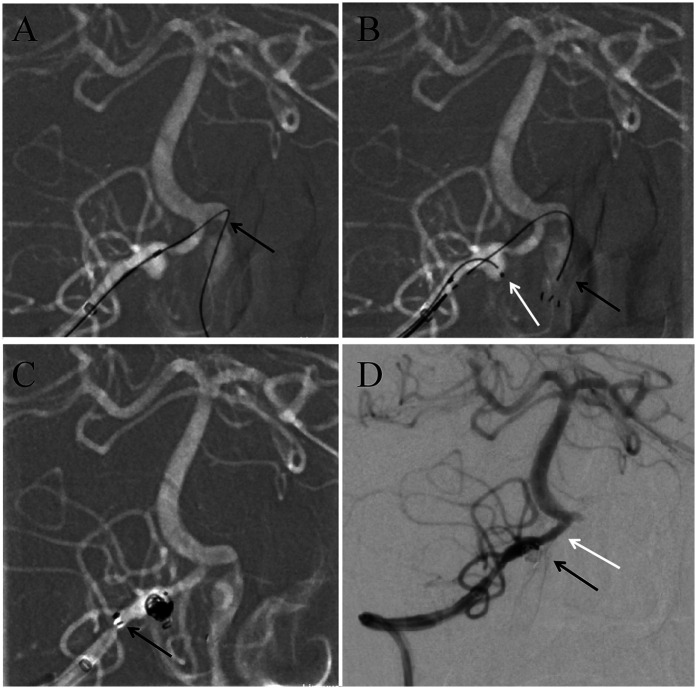
**(A)** Intraoperative imaging showed the microguide wire carryied the stent microcatheter cross over the vertebrobasilar artery junction to reach the contralateral vertebral artery V4 segment. **(B)** The black arrow showed the Enterprise-2 stent was open and well attached to the vessel wall. The white arrow showed the SL-10 microcatheter entered the lumen of the dissecting aneurysm. **(C)** showed complete release of the stent after aneurysm embolization. This black arrow indicated that the end of the sent was fully open. **(D)** Digital subtraction angiography (DSA) examination was performed again after operation. The black arrow indicated that the aneurysm embolization was complete. The white arrow indicated a significant improvement in the stenosis of the distal vertebral artery.

**Figure 3:**
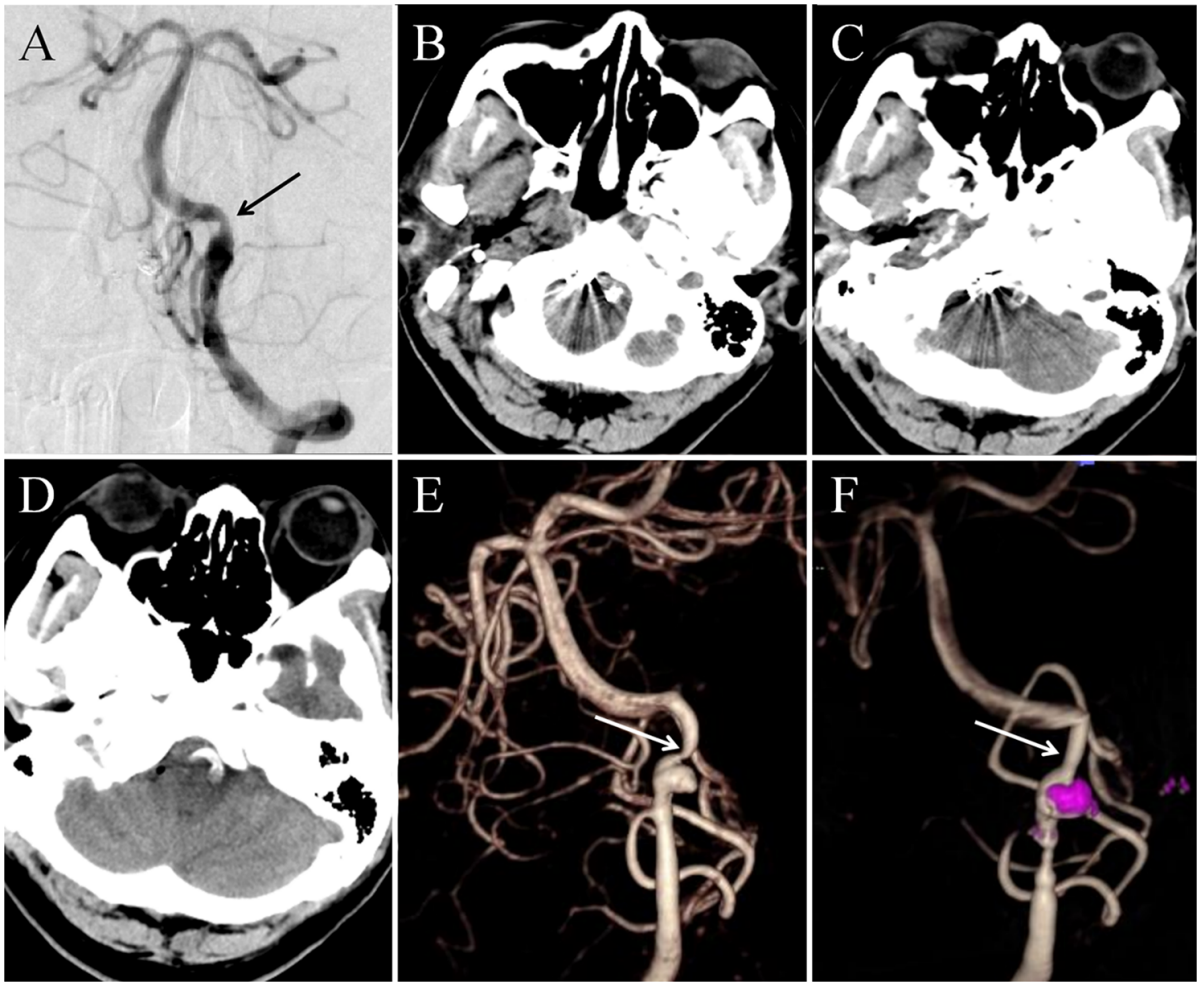
**(A)** Postoperative angiography indicated that the distal stenosis of the contralateral vertebral artery had been relieved. **(B–D)** Postoperative head CT examination revealed that the stent crossed the V4 segment of the bilateral vertebral artery, and no new hemorrhage and cerebral infarction were found. **(E,F)** Preoperative and postoperative 3D reconstruction showed that vascular stenosis was significantly improved, the single stent completely covered the dissection and stenosis of bilateral vertebral arteries, and basilar aneurysm blood flow was unobstructed.

Oral antiplatelet therapy was given 2 h before surgery with 300 mg of aspirin and clopidogrel respectively. The patient underwent endovascular interventional therapy on May 16, 2024. After successful general anesthesia, disinfection and sterile surgical sheets were routinely performed. The right femoral artery was punctured using the Seldinger technique and a 6F sheath was inserted. Then connect the Y-valve, tee and pressure injector. An 6F intermediate guiding catheter (Tethys®; Peijia Medical Technology, Suzhou, CNA) was placed through the Y valve and carefully advanced under fluoroscopy to reach the right truncus brachiocephalicus. Under the Roadmap, the intermediate guiding catheter was continued to be pushed to the V2 level of the right vertebral artery. A 205 cm NeuroScout microguide wire (NeuroScout steerable guidewire; Codman, Massachusetts, USA) was used to carry stent microcatheter (Prowler Select Plus; Coman Corporation, Chaska, MN, USA) across the confluence of the vertebrobasilar artery into the V2 segment of left vertebral artery (Figure 2A). An Enterprise-2 stent (4.0mm × 39 mm; Coman Corporation, Chaska, MN, USA) was slowly delivered through the stent microcatheter and carefully partly released after it was in place (Figure 2B). The NeuroScout microguide wire was then used to carry another microcatheter (Excelsior SL-10; Boston Scientific Corporation, Fremont, CA, USA) into the vertebral artery through the Y valve, and this SL-10 microcatheter was then superselected into the aneurysm under the guidance of Roadmap (Figure 2B). The head end of the single stent covered the proximal end of the left vertebral artery dilated dissection, and the tail end of the stent covered the proximal end of the right vertebral artery ruptured dissecting aneurysm, ensuring the full coverage of the neck of the right ruptured aneurysm, two severe stenosis of the vertebral artery and the left vertebral artery dissection. Four appropriately sized coils (2.0 mm × 8.0 cm × 2, 3.0 mm × 8.0 cm × 2; NRcoil™, Peijia Medical Technology, Suzhou, CNA) were gradually inserted into the aneurysm by SL-10 microcatheter until the aneurysm was radiographically completely occlusive (Figure 2C). Postoperative right vertebral arteriography showed that the ruptured dissected artery was densely embolized, and the vertebrobasilar artery flow was not affected. The right vertebral artery was clearly displayed, the stent was well attached to the vascular wall, and the stenosis at the end of the right vertebral artery was significantly relieved (Figure 2D). Anterior and lateral imaging of the left vertebral artery also showed that the stent was well attached to the wall, and additional distal stenosis was also improved (Figure 3A). The patient was awake after operation and speech and limb movement were normal. Head CT after operation showed no new hemorrhage or cerebral infarction (Figures 3B–D). Preoperative and postoperative 3D reconstruction showed that vascular stenosis was significantly improved, the single stent completely covered the dissection and stenosis of bilateral vertebral arteries, and basilar aneurysm blood flow was unobstructed (Figures 3E,F).

## 4 Discussion

The formation of intracranial vertebral artery dissection was associated with atherosclerosis (5). The incidence of intracranial vertebral artery dissection was high in Asian population due to the high incidence of intracranial atherosclerosis (1). Vertebral artery dissection was usually formed between the elastic membrane in the blood wall of the vertebral artery and the media (4). The media and outer membrane of the intracranial vertebral artery was thin and after the formation of the dissection, it was easy to progress outwards, which at last forming VADAs and leading to SAH (13). The early rebleeding rate of ruptured VADAs was very high, and active treatment is needed in the early stage of the disease (14). Due to the disability and high mortality caused by surgical treatment in the acute stage, endovascular interventional therapy has gradually become the first choice of treatment (15).

In two cases, the dissecting aneurysm was occlusioned and a vessel bypass surgery was performed, which resulted in mild ischemia. Transient thrombosis occurred in two cases of stent assisted aneurysm embolization. The last case, a pediatric patient, was treated conservatively. As the patient's condition was stable, regular follow-up was decided. Therefore, at present the main therapeutic method of VADA was endovascular interventional therapy, including occlusive therapy and endovascular reconstruction therapy (15). Occlusive therapy should ensure sufficient blood flow compensation after occlusion, otherwise it may lead to the occurrence of large cerebral infarction (16). The endovascular reconstruction of VADA mainly included single stent-assisted coil embolization, overlapping stent placement and flow diverter (FD) placement (17).

In this case, the condition of bilateral vertebral artery vascular was extremely special. Right vertebral artery dissection aneurysm was the focal point of hemorrhage, and the distal ipilateral vertebral artery was associated with severe stenosis. In addition, there were unruptured dissecting aneurysm and severe distal stenosis in the intracranial segment of the left vertebral artery. This complex case was discussed in detail in our joint treatment center before surgery. If overlapping stent placement or FD placement were used, this ruptured dissecting aneurysm could be resolved. However, the severe stenosis of the blood vessels at the end of the ipsilateral vertebral artery might hinder the release and adherence of the stent, thus affecting the blood flow of the vertebral artery or the puncture vessel and resulting in cerebral infarction. In addition, it has been reported that the application of FD placement had high requirements for perioperative antiplatelet therapy, which might increase the risk of re-rupture of dissecting aneurysm. The patient's family had limited financial resources and could not afford expensive blood flow guidance devices. Furthermore, the release of FD could not prophylactically address the problems of contralateral vertebral artery dissection and vessel stenosis. Therefore, the endovascular reconstruction treatment strategy in this case became a thorny issue.

Creatively using the Transmountain technique, a single Enterprise-2 stent was released to simultaneously cover the neck of the ruptured dissecting aneurysm on the right, the unruptured dilated vascular dissecting on the left, and the two sites of severe vascular stenosis. In this case, the single stent played four important roles at the same time, including assisting the coil embolization of the wide-necked ruptured aneurysm, covering the left vertebral artery dissection, supporting the right vertebral artery terminal severe stenosis, and supporting the left vertebral artery terminal severe stenosis. Moreover, because Enterpreise-2 stent was a laser sculpting stent with low mesh metal coverage, the vertebrobasilar artery flow was not affected after vertebrobasilar junction was covered. Therefore, this single stent not only could assist the embolization of the ruptured aneurysm, but also could improve the severe stenosis of the ipsilateral distal blood vessel. Additionally, the 39 mm Enterpreise-2 stent could also cover the dilatation of the contralateral vertebral artery preventively, preventing the dissection from becoming larger and causing rupture or narrowing of the vessel. All in all, a single interventional operation completely solved the multiple lesions of the patient's blood vessels, which greatly reducing the pain of the patient and saving huge medical costs for the patient who was not wealthy.

In this case we used a single stent to reconstruct both vertebral arteries. This procedure saved the patient a lot of financial burden. At the same time, the release of the stent in the vertebral artery on the side of the aneurysm lesion could effectively assist the embolization of the aneurysm. Immediate angiography showed that the satisfaction of aneurysm embolization was more than 95%. Frankly, the stability of embolism still needed to be determined by further follow-up review. But at least for now we've solved a life-threatening bleeding problem with this method. In addition, we reconstructed the contralateral narrow vertebral artery by means of a transmountain stent release, which expanded the vertebral artery diameter and provided compensatory support for the blood supply to the posterior circulation. Furthermore, contralateral vascular reconstruction could also prevent the formation of dissection aneurysm. Therefore, on the basis of the lowest economic burden, our operation not only solved the problem of the responsible lesion, but also played an important role in the prevention of various lesions.

Some medical centers reported that the application of overlapping stents could eliminate the vascular dissection better than the single stent-assisted coil embolization by reducing the mesh area of the stent (7). It has also been reported that more and more treatment centers are using FD to treat dissection aneurysms (3). The central idea of applying these two techniques was to improve the metal coverage of the interlayer, reduce the blood flow into the tumor, and promote the healing of the aneurysm (18). In contrast to the surgical approaches employed by other institutions, our center adheres to the principle of “If it can be simple, do not complicate”. Guided by this surgical philosophy, we are often able to minimize operation time, thereby alleviating the financial burden on patients' families and most importantly, reducing surgical risks.

However, there was no unified standard for the treatment of endovascular reconstruction in VADA, and the choice of treatment mainly depended on the location and morphology of the aneurysm and the relationship between the aneurysm and the collateral circulation vessels (19). According to different cases, more accurate individual treatment strategies should be selected, and the complications and treatment costs should be reduced as much as possible.

## 5 Limitation

Our article does have the following shortcomings. First of all, the summary and statistics of the literature are not detailed enough to systematically summarize the disability rate and mortality rate in the case information. Second, the treatment method of the cases we provided is so special that it is sometimes difficult to promote this method in clinic. Finally, because there are few cases of multi-center vertebrobasilar aneurysms, it is difficult to make detailed statistics on aneurysm occlusion, stenosis and mortality. These are limitations of our paper, and we will continue to summarize the clinical treatment experience of vertebral artery dissection aneurysm and improve our data analysis ability.

## 6 Conclusion

Although vertebral artery dissection aneurysm with complex vascular conditions like this was rare, we creatively provided a novel endovascular treatment strategy. This method of stent release not only assisted the embolization of ruptured aneurysm, but also prevented the progression of unruptured vascular dissection and improved the severe stenosis of bilateral vertebral artery, which providing a new idea for the treatment of intracranial vertebral artery dissected aneurysm.

## Data Availability

The datasets presented in this article are not readily available because of ethical and privacy restrictions. Requests to access the datasets should be directed to the corresponding authors.

## Supplementary material

Supplementary Figure S1 Flow chart for literature screening and inclusion.
