# Supplementary figures and images for: Stent-assisted coil embolization of ruptured vertebral artery dissected aneurysm with severe stenosis of bilateral vertebral artery V4 segment by the transmountain technique: a case report and review of the literatures
Source: Front Surg. 2025 Feb 17;12:1442122. doi: 10.3389/fsurg.2025.1442122 (PMC11872894; doi:10.3389/fsurg.2025.1442122)

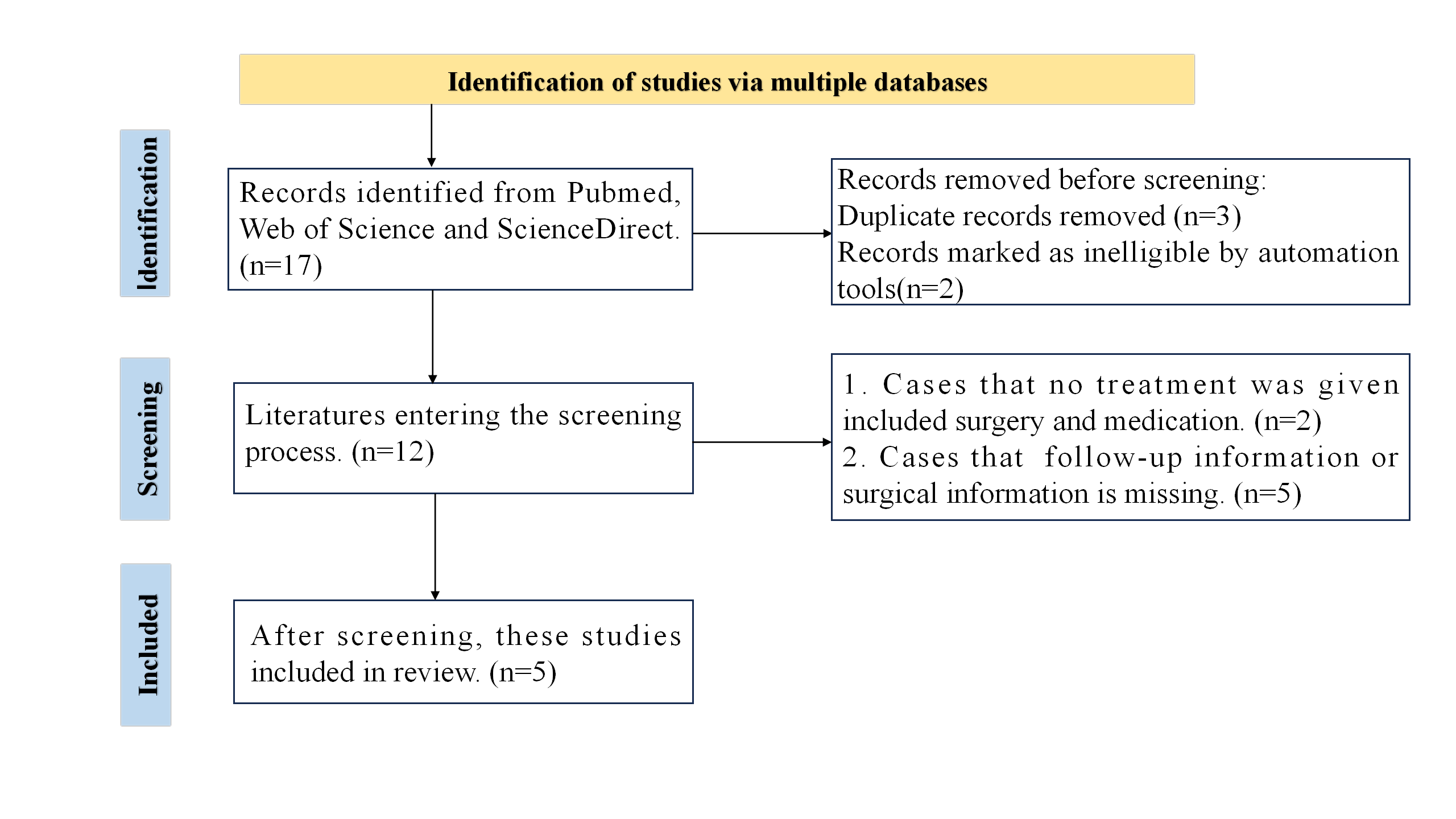

Supplement: Supplementary Figure S1 [file Image1.tif]
